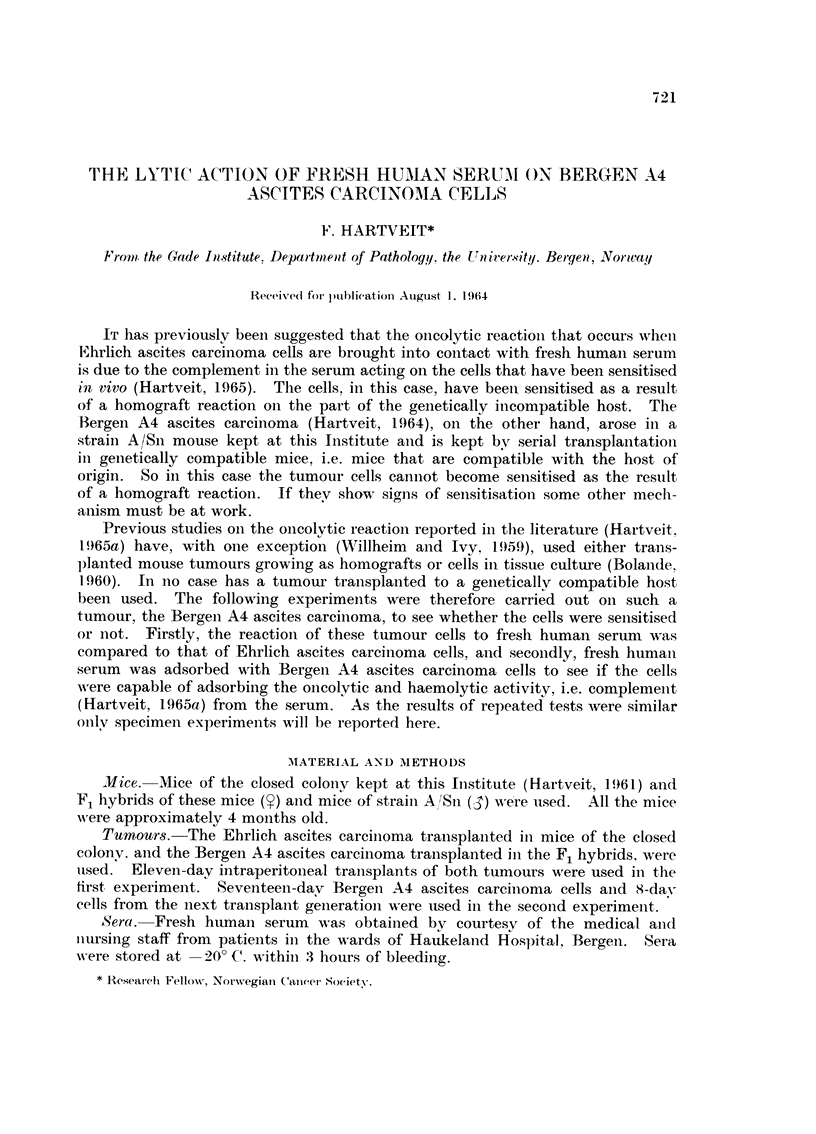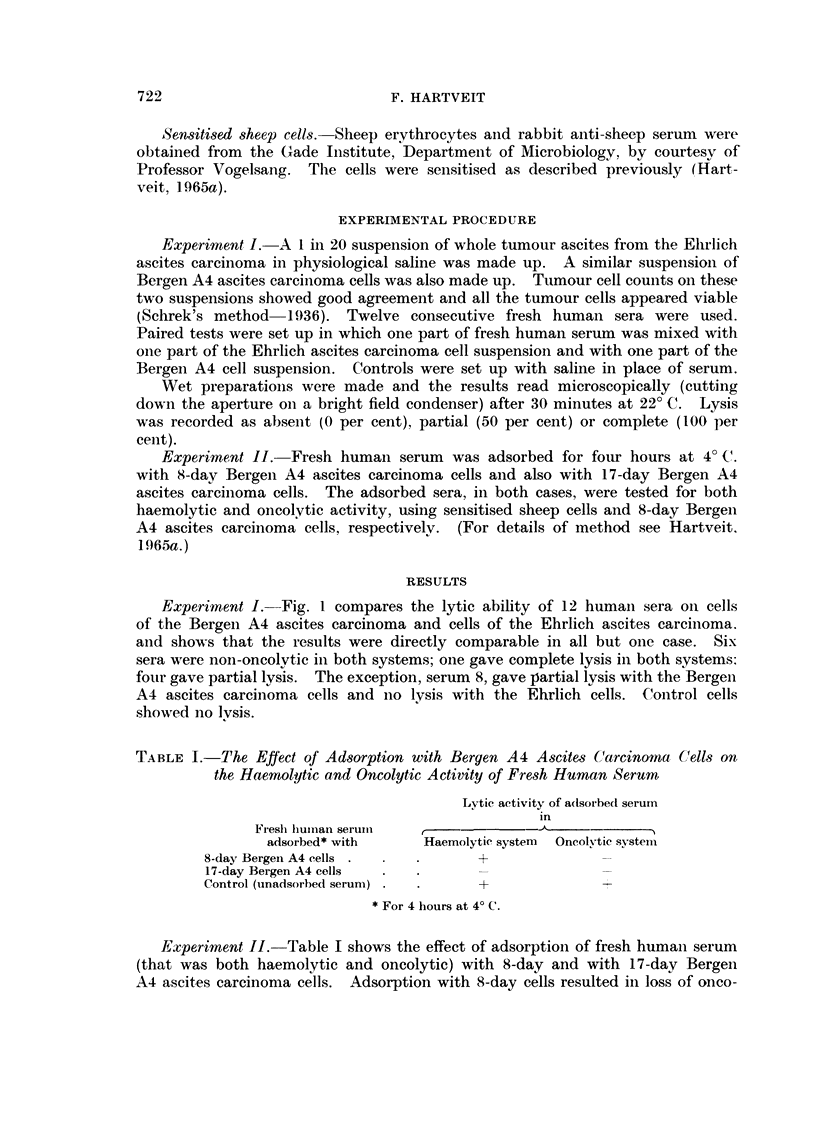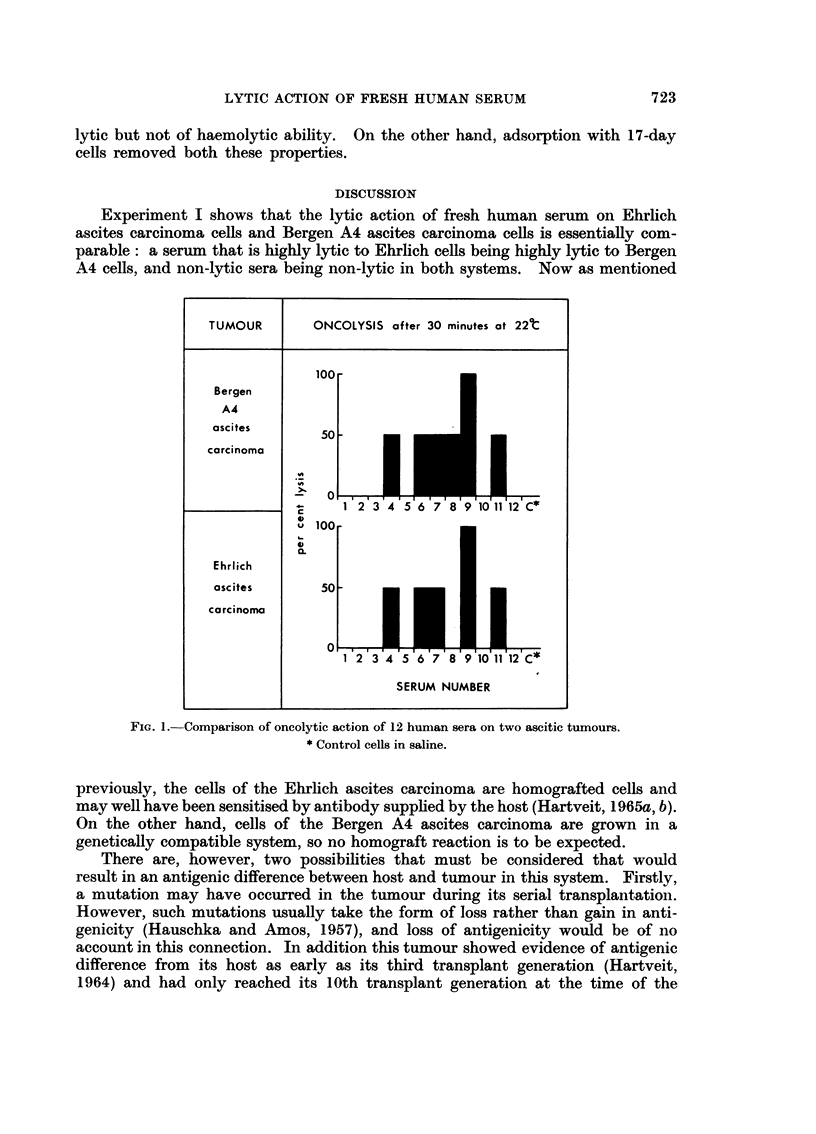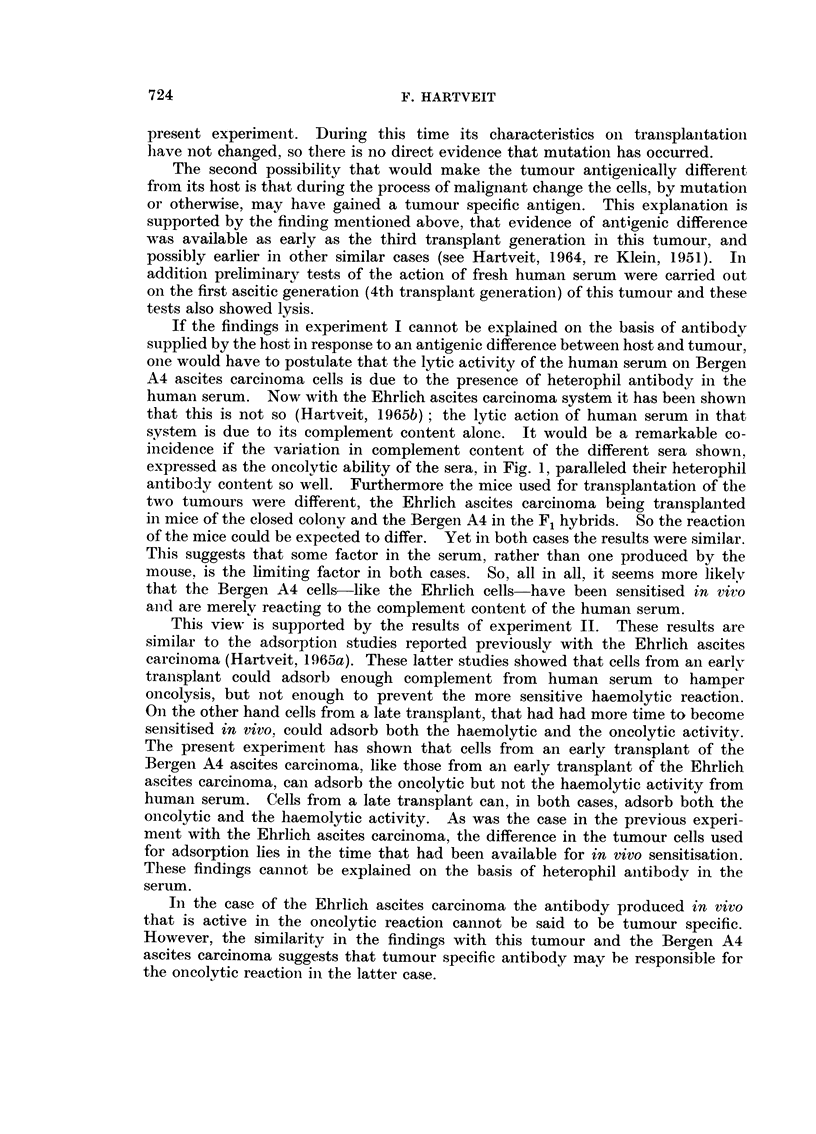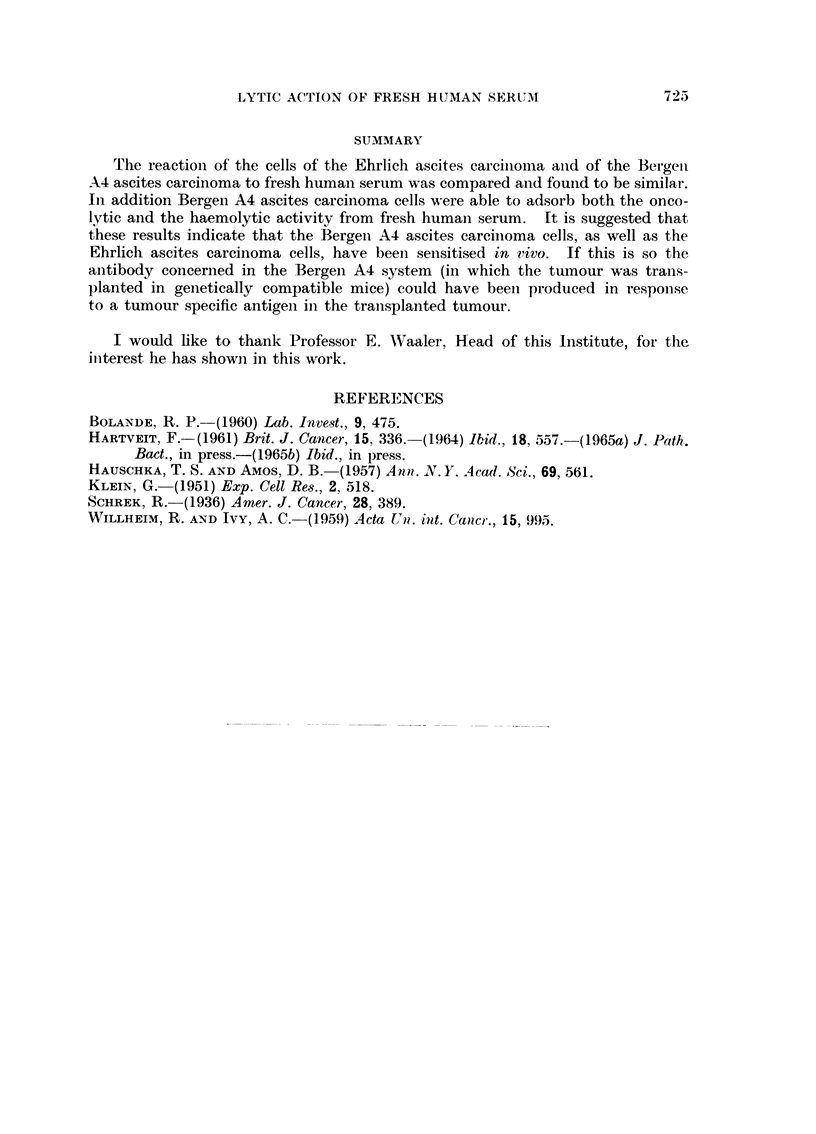# The Lytic Action of Fresh Human Serum on Bergen A4 Ascites Carcinoma Cells

**DOI:** 10.1038/bjc.1964.83

**Published:** 1964-12

**Authors:** F. Hartveit


### THE LYTIC ACTION OF FRESH HUMAN SERUM ON BERGEN A4 ASCITES CARCINOMA CELLS

F. HARTVEIT*

*From the Gade Institute, Department of Pathology, the University, Bergen, Norway*

Received for publication August 1, 1964

IT has previously been suggested that the oncolytic reaction that occurs when Ehrlich ascites carcinoma cells are brought into contact with fresh human serum is due to the complement in the serum acting on the cells that have been sensitised *in vivo* (Hartveit, 1965). The cells, in this case, have been sensitised as a result of a homograft reaction on the part of the genetically incompatible host. The Bergen A4 ascites carcinoma (Hartveit, 1964), on the other hand, arose in a strain A/Sn mouse kept at this Institute and is kept by serial transplantation in genetically compatible mice, i.e. mice that are compatible with the host of origin. So in this case the tumour cells cannot become sensitised as the result of a homograft reaction. If they show signs of sensitisation some other mechanism must be at work.

Previous studies on the oncolytic reaction reported in the literature (Hartveit, 1965*a*) have, with one exception (Willheim and Ivy, 1959), used either transplanted mouse tumours growing as homografts or cells in tissue culture (Bolande, 1960). In no case has a tumour transplanted to a genetically compatible host been used. The following experiments were therefore carried out on such a tumour, the Bergen A4 ascites carcinoma, to see whether the cells were sensitised or not. Firstly, the reaction of these tumour cells to fresh human serum was compared to that of Ehrlich ascites carcinoma cells, and secondly, fresh human serum was adsorbed with Bergen A4 ascites carcinoma cells to see if the cells were capable of adsorbing the oncolytic and haemolytic activity, i.e. complement (Hartveit, 1965*a*) from the serum. As the results of repeated tests were similar only specimen experiments will be reported here.

### MATERIAL AND METHODS

*Mice.*—Mice of the closed colony kept at this Institute (Hartveit, 1961) and $F_1$ hybrids of these mice (♀) and mice of strain A/Sn (♂) were used. All the mice were approximately 4 months old.

*Tumours.*—The Ehrlich ascites carcinoma transplanted in mice of the closed colony, and the Bergen A4 ascites carcinoma transplanted in the $F_1$ hybrids, were used. Eleven-day intraperitoneal transplants of both tumours were used in the first experiment. Seventeen-day Bergen A4 ascites carcinoma cells and 8-day cells from the next transplant generation were used in the second experiment.

*Sera.*—Fresh human serum was obtained by courtesy of the medical and nursing staff from patients in the wards of Haukeland Hospital, Bergen. Sera were stored at $-20°$ C. within 3 hours of bleeding.

* Research Fellow, Norwegian Cancer Society.

*Sensitised sheep cells.*—Sheep erythrocytes and rabbit anti-sheep serum were obtained from the Gade Institute, Department of Microbiology, by courtesy of Professor Vogelsang. The cells were sensitised as described previously (Hartveit, 1965*a*).

### EXPERIMENTAL PROCEDURE

*Experiment I.*—A 1 in 20 suspension of whole tumour ascites from the Ehrlich ascites carcinoma in physiological saline was made up. A similar suspension of Bergen A4 ascites carcinoma cells was also made up. Tumour cell counts on these two suspensions showed good agreement and all the tumour cells appeared viable (Schrek's method—1936). Twelve consecutive fresh human sera were used. Paired tests were set up in which one part of fresh human serum was mixed with one part of the Ehrlich ascites carcinoma cell suspension and with one part of the Bergen A4 cell suspension. Controls were set up with saline in place of serum.

Wet preparations were made and the results read microscopically (cutting down the aperture on a bright field condenser) after 30 minutes at 22° C. Lysis was recorded as absent (0 per cent), partial (50 per cent) or complete (100 per cent).

*Experiment II.*—Fresh human serum was adsorbed for four hours at 4° C. with 8-day Bergen A4 ascites carcinoma cells and also with 17-day Bergen A4 ascites carcinoma cells. The adsorbed sera, in both cases, were tested for both haemolytic and oncolytic activity, using sensitised sheep cells and 8-day Bergen A4 ascites carcinoma cells, respectively. (For details of method see Hartveit, 1965*a*.)

### RESULTS

*Experiment I.*—Fig. 1 compares the lytic ability of 12 human sera on cells of the Bergen A4 ascites carcinoma and cells of the Ehrlich ascites carcinoma, and shows that the results were directly comparable in all but one case. Six sera were non-oncolytic in both systems; one gave complete lysis in both systems; four gave partial lysis. The exception, serum 8, gave partial lysis with the Bergen A4 ascites carcinoma cells and no lysis with the Ehrlich cells. Control cells showed no lysis.

TABLE I.—*The Effect of Adsorption with Bergen A4 Ascites Carcinoma Cells on the Haemolytic and Oncolytic Activity of Fresh Human Serum*

| Fresh human serum adsorbed* with | Lytic activity of adsorbed serum in: Haemolytic system | Oncolytic system |
|---|---|---|
| 8-day Bergen A4 cells . . . | + | — |
| 17-day Bergen A4 cells . . | — | — |
| Control (unadsorbed serum) . . | + | + |

* For 4 hours at 4° C.

*Experiment II.*—Table I shows the effect of adsorption of fresh human serum (that was both haemolytic and oncolytic) with 8-day and with 17-day Bergen A4 ascites carcinoma cells. Adsorption with 8-day cells resulted in loss of oncolytic but not of haemolytic ability. On the other hand, adsorption with 17-day cells removed both these properties.

### DISCUSSION

Experiment I shows that the lytic action of fresh human serum on Ehrlich ascites carcinoma cells and Bergen A4 ascites carcinoma cells is essentially comparable : a serum that is highly lytic to Ehrlich cells being highly lytic to Bergen A4 cells, and non-lytic sera being non-lytic in both systems. Now as mentioned

FIG. 1.—Comparison of oncolytic action of 12 human sera on two ascitic tumours.
* Control cells in saline.

previously, the cells of the Ehrlich ascites carcinoma are homografted cells and may well have been sensitised by antibody supplied by the host (Hartveit, 1965*a*, *b*). On the other hand, cells of the Bergen A4 ascites carcinoma are grown in a genetically compatible system, so no homograft reaction is to be expected.

There are, however, two possibilities that must be considered that would result in an antigenic difference between host and tumour in this system. Firstly, a mutation may have occurred in the tumour during its serial transplantation. However, such mutations usually take the form of loss rather than gain in antigenicity (Hauschka and Amos, 1957), and loss of antigenicity would be of no account in this connection. In addition this tumour showed evidence of antigenic difference from its host as early as its third transplant generation (Hartveit, 1964) and had only reached its 10th transplant generation at the time of the present experiment. During this time its characteristics on transplantation have not changed, so there is no direct evidence that mutation has occurred.

The second possibility that would make the tumour antigenically different from its host is that during the process of malignant change the cells, by mutation or otherwise, may have gained a tumour specific antigen. This explanation is supported by the finding mentioned above, that evidence of antigenic difference was available as early as the third transplant generation in this tumour, and possibly earlier in other similar cases (see Hartveit, 1964, re Klein, 1951). In addition preliminary tests of the action of fresh human serum were carried out on the first ascitic generation (4th transplant generation) of this tumour and these tests also showed lysis.

If the findings in experiment I cannot be explained on the basis of antibody supplied by the host in response to an antigenic difference between host and tumour, one would have to postulate that the lytic activity of the human serum on Bergen A4 ascites carcinoma cells is due to the presence of heterophil antibody in the human serum. Now with the Ehrlich ascites carcinoma system it has been shown that this is not so (Hartveit, 1965*b*) ; the lytic action of human serum in that system is due to its complement content alone. It would be a remarkable coincidence if the variation in complement content of the different sera shown, expressed as the oncolytic ability of the sera, in Fig. 1, paralleled their heterophil antibody content so well. Furthermore the mice used for transplantation of the two tumours were different, the Ehrlich ascites carcinoma being transplanted in mice of the closed colony and the Bergen A4 in the $F_1$ hybrids. So the reaction of the mice could be expected to differ. Yet in both cases the results were similar. This suggests that some factor in the serum, rather than one produced by the mouse, is the limiting factor in both cases. So, all in all, it seems more likely that the Bergen A4 cells—like the Ehrlich cells—have been sensitised *in vivo* and are merely reacting to the complement content of the human serum.

This view is supported by the results of experiment II. These results are similar to the adsorption studies reported previously with the Ehrlich ascites carcinoma (Hartveit, 1965*a*). These latter studies showed that cells from an early transplant could adsorb enough complement from human serum to hamper oncolysis, but not enough to prevent the more sensitive haemolytic reaction. On the other hand cells from a late transplant, that had had more time to become sensitised *in vivo*, could adsorb both the haemolytic and the oncolytic activity. The present experiment has shown that cells from an early transplant of the Bergen A4 ascites carcinoma, like those from an early transplant of the Ehrlich ascites carcinoma, can adsorb the oncolytic but not the haemolytic activity from human serum. Cells from a late transplant can, in both cases, adsorb both the oncolytic and the haemolytic activity. As was the case in the previous experiment with the Ehrlich ascites carcinoma, the difference in the tumour cells used for adsorption lies in the time that had been available for *in vivo* sensitisation. These findings cannot be explained on the basis of heterophil antibody in the serum.

In the case of the Ehrlich ascites carcinoma the antibody produced *in vivo* that is active in the oncolytic reaction cannot be said to be tumour specific. However, the similarity in the findings with this tumour and the Bergen A4 ascites carcinoma suggests that tumour specific antibody may be responsible for the oncolytic reaction in the latter case.

### SUMMARY

The reaction of the cells of the Ehrlich ascites carcinoma and of the Bergen A4 ascites carcinoma to fresh human serum was compared and found to be similar. In addition Bergen A4 ascites carcinoma cells were able to adsorb both the oncolytic and the haemolytic activity from fresh human serum. It is suggested that these results indicate that the Bergen A4 ascites carcinoma cells, as well as the Ehrlich ascites carcinoma cells, have been sensitised *in vivo*. If this is so the antibody concerned in the Bergen A4 system (in which the tumour was transplanted in genetically compatible mice) could have been produced in response to a tumour specific antigen in the transplanted tumour.

I would like to thank Professor E. Waaler, Head of this Institute, for the interest he has shown in this work.

### REFERENCES

BOLANDE, R. P.—(1960) *Lab. Invest.*, **9**, 475.
HARTVEIT, F.—(1961) *Brit. J. Cancer*, **15**, 336.—(1964) *Ibid.*, **18**, 557.—(1965*a*) *J. Path. Bact.*, in press.—(1965*b*) *Ibid.*, in press.
HAUSCHKA, T. S. AND AMOS, D. B.—(1957) *Ann. N.Y. Acad. Sci.*, **69**, 561.
KLEIN, G.—(1951) *Exp. Cell Res.*, **2**, 518.
SCHREK, R.—(1936) *Amer. J. Cancer*, **28**, 389.
WILLHEIM, R. AND IVY, A. C.—(1959) *Acta Un. int. Cancr.*, **15**, 995.